# Manipulating Atomic Disorder and Mesoscale Architectures for High‐Efficiency Thermoelectric Modules

**DOI:** 10.1002/advs.74899

**Published:** 2026-05-07

**Authors:** Jiwu Xin, Bo Wang, Chengyun Xu, Wang Li, Pengyu Zhang, Dongwang Yang, Yongke Wang, Jinfeng Dong, Lei Wei, Ting Zhang, Qinghui Jiang

**Affiliations:** ^1^ State Key Laboratory of Materials Processing and Die and Mould Technology Huazhong University of Science and Technology Wuhan P. R. China; ^2^ School of Electrical and Electronic Engineering Nanyang Technological University Singapore Singapore; ^3^ Hangzhou International Innovation Institute Beihang University Hangzhou P. R. China; ^4^ Institute of Engineering Thermophysics Chinese Academy of Sciences Beijing P. R. China; ^5^ School of Materials Science and Engineering Nanyang Technological University Singapore Singapore; ^6^ Engineering Department University of Miami Miami USA; ^7^ University of Chinese Academy of Sciences Nanjing P. R. China; ^8^ School of Microelectronics Wuhan Textile University Wuhan China

**Keywords:** atomic disorder, bismuth telluride, in situ reaction, mesoscale architectures, thermoelectric module

## Abstract

Decoupling electron and phonon transport remains the central challenge in designing high‐performance thermoelectric materials for low‐grade heat recovery. Conventional optimization strategies for Bi_2_Te_3_‐based alloys typically isolate defect engineering from microstructure modulation, often leading to performance trade‐offs. Here, we demonstrate a strategy that simultaneously manipulates atomic disorder and mesoscale architectures via an in situ solid‐state reaction in p‐type Bi_0.5_Sb_1.5_Te_3_. By incorporating PbTiO_3_ precursors, we trigger a thermodynamically driven reaction: at the atomic scale, Pb atoms substitutionally occupy Sb sites to induce chemical disorder and optimize the Fermi level; at the mesoscale, residual Ti species segregate to form PbTiO_3_@TiO_2_ core–shell precipitates. This hierarchical structural engineering acts as a frequency‐selective barrier, drastically reducing lattice thermal conductivity without compromising carrier mobility. Consequently, the 0.5 mol% composite achieves a peak figure of merit of 1.47 at 333 K. Translating this material‐level breakthrough into a device, we fabricated a thermoelectric module that delivers a conversion efficiency of ∼7% and an output power of 13.1 mW under a temperature difference of 180 K. This work establishes a generalizable protocol for functionalizing thermoelectric systems via coupled defect chemistry and interface engineering, bridging the gap between fundamental transport physics and practical energy harvesting.

## Graphical abstract

The in situ reaction of PbTiO_3_ precursor reacts in situ with the Bi_0.5_Sb_1.5_Te_3_ matrix, effectively enlarging the energy barrier for minority carrier excitation and suppressing bipolar diffusion, and predominantly scattering mid‐to‐low frequency phonons. Ultimately, a single thermoelectric module was fabricated to achieve a competitive efficiency of ∼7% at 180 K temperature difference.

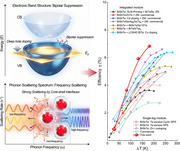

## 1 Introduction

Low‐grade waste heat recovery represents a pivotal frontier in sustainable energy technologies, offering a pathway to decarbonize the global energy landscape. Among the candidates for near‐room‐temperature harvesting, Bi_2_Te_3_‐based thermoelectric materials—particularly p‐type Bi_0.5_Sb_1.5_Te_3_ (BST)—stand as the commercial benchmark. The conversion efficiency of these materials is governed by the dimensionless figure of merit, *zT = S^2^σT*/(*κ*_e_+*κ*_ph_) [1, 2, 3, 4, 5, 6], where the interdependent nature of the Seebeck coefficient (*S*), electrical conductivity (*σ*), and electronic thermal conductivity (*κ*_e_) creates a formidable bottleneck [6, 7, 8, 9, 10, 11, 12]. Conventional optimization strategies have largely oscillated between two extremes: introducing dopants (e.g., Ag [13], Cu [14], Cd [15], Pb [16], and Mn [17]) to tune the carrier concentration, or embedding insulating oxides (e.g., Ta_2_O_5_ [18], SiO_2_ [19], Al_2_O_3_ [20], ZnO [21], ZrO_2_ [22], and Y_2_O_3_ [23]) to scatter phonons. However, these independent approaches are often mutually exclusive; dopants frequently fail to suppress lattice thermal conductivity (*κ*_ph_), while physically mixed oxide inclusions tend to agglomerate and introduce incoherent interfaces that severely degrade carrier mobility.

To circumvent these trade‐offs, the concept of in situ solid‐state reaction has emerged as a compelling paradigm. Unlike ex situ mechanical mixing, thermodynamically driven solid‐state reactions can spontaneously generate second phases with atomically clean, coherent interfaces, thereby minimizing extrinsic carrier scattering while maximizing phonon impedance. This approach offers a unique opportunity for “synergistic modulation”: using a single precursor to simultaneously trigger electronic doping and nanostructural evolution during the sintering process.

**FIGURE 1:**
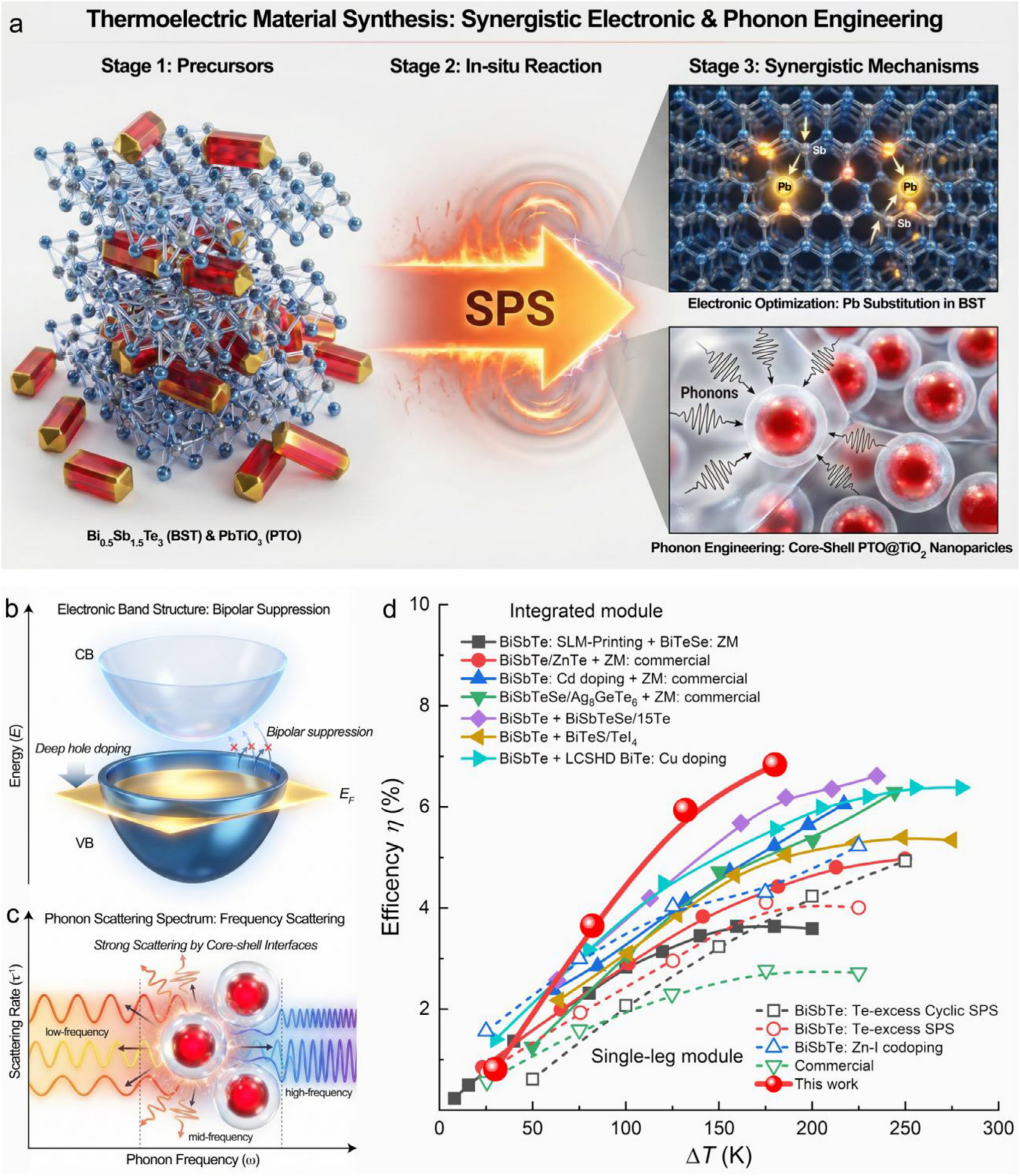
Manipulating atomic disorder and mesoscale architectures for high‐performance thermoelectrics. (a), Schematic illustration of the in situ reaction strategy. During spark plasma sintering (SPS), the (PTO) precursor reacts in situ with the (BST) matrix: Pb atoms substitute Sb sites (electronic optimization), while residual Ti species segregate to form PTO@TiO_2_ core–shell nanostructures at grain boundaries (phonon engineering). (b), The physical mechanism of transport decoupling. Schematic electronic band structure showing that Pb doping pushes the Fermi level deep into the valence band (VB), effectively enlarging the energy barrier for minority carrier excitation and intrinsically suppressing bipolar diffusion. (c), Phonon frequency spectrum illustrating that the in situ formed multiscale core–shell interfaces act as frequency‐selective barriers, predominantly scattering mid‐to‐low frequency phonons. (d), Comparison of the energy conversion efficiency   of the PTO/BST module in this work against state‐of‐the‐art Bi_2_Te_3_‐based modules reported in the literature over the past decade. The fabricated single‐stage thermoelectric module achieves a competitive efficiency of ∼7% at a temperature difference (ΔT) of 180 K.

Here, we report a holistic strategy that integrates atomic disorder engineering with mesoscale structural modulation by introducing perovskite PbTiO_3_ (PTO) into the BST matrix, as conceptually illustrated in Figure 1a. We leverage the intrinsic reactivity between PTO and BST to orchestrate a “one‐stone‐two‐birds” effect: (1) Atomic disorder: Pb atoms diffuse into the lattice to occupy Sb sites, optimizing the hole concentration and pushes the Fermi level (E_F_) deep into the valence band, intrinsically suppressing bipolar diffusion (Figure 1b). (2) Mesoscale architectures: Residual Ti species segregate to form core‐shell PTO@TiO_2_ architectures at the grain boundaries, validating that manipulating structural features across length scales is a generalizable route to act as a frequency‐selective barrier, drastically reducing *κ*_ph_ by scattering mid‐to‐low frequency phonons without compromising electrical integrity (Figure 1c). Consequently, we achieved a peak *zT* of 1.47 at 333 K. Distinct from previous works, we successfully translated this material‐level breakthrough into a practical device. The fabricated thermoelectric module demonstrates a conversion efficiency of ∼7%, establishing a competitive performance among state‐of‐the‐art Bi_2_Te_3_‐based technologies [24, 25, 26, 27, 28, 29, 30, 31, 32] (Figure 1d).

## 2 Materials and Methods

### 2.1 Materials Synthesis

BST was weighed in stoichiometric proportions (Bi, 99.999%; Sb, 99.999%; Te, 99.999%) and subjected to mechanical alloying. The ingots were pulverized and sieved (200 mesh) to obtain fine powders. Commercial PTO powders (<5 µm, Sigma‐Aldrich) were added to the BST matrix at varying molar ratios (*x* = 0–1.5 mol%). The mixture was mechanically alloyed in stainless‐steel jars under an argon atmosphere using a planetary ball mill (ball‐to‐powder weight ratio of 20:1) at 280 rpm for 2 h. The resulting composite powders were consolidated via SPS at 723 K for 8 min under a uniaxial pressure of 60 MPa in a vacuum.

### 2.2 Structural Characterization

Phase identification was performed using X‐ray diffraction (XRD; XRD‐7000, Shimadzu) with Cu Kα radiation. The microstructures and elemental distributions were analyzed using field‐emission scanning electron microscopy (FE‐SEM; GeminiSEM 300, Carl Zeiss) equipped with energy‐dispersive X‐ray spectroscopy (EDS). Atomic‐scale structural features and lattice defects were characterized by high‐resolution transmission electron microscopy (HR‐TEM; Tecnai G2 F30, FEI).

### 2.3 Thermoelectric Transport Measurement

Electrical transport properties (resistivity *ρ* and Seebeck coefficient *S*) were measured simultaneously using a commercial system (ZEM‐3, ULVAC‐Riko) under a helium atmosphere from 298 to 513 K. The Hall coefficient (*R*_H_) was determined using a Hall measurement system (HMS‐5500, ECOPIA) under a 0.55 T magnetic field. The carrier concentration *n* and mobility (*μ*) were calculated via *n*_H_ = 1/(e*R*_H_) and *μ* = 1/(e*n*_H_ρ), respectively. Thermal conductivity (*κ*) was calculated using *κ* = D*ρC*_p_. The thermal diffusivity (*D*) was measured using the laser flash method (LFA‐427, NETZSCH) on graphite‐coated disk samples. The specific heat capacity (*C*_p_) was estimated using the Debye model [33] (details in Note S2), and the density (*d*) was determined by the Archimedes method. The combined uncertainty for the *zT* calculation is estimated to be ∼12%.

### 2.4 Module Fabrication and Evaluation

A thermoelectric unicouple was assembled using the optimized p‐type BST/0.5%PTO composite (height: 13.5 mm; cross‐sectional area: 11.84 mm^2^) and an n‐type Bi_2_Te_2.7_Se_0.3_ leg (height: 13.5 mm; cross‐sectional area: 8.91 mm^2^). To minimize contact resistance, Ni diffusion barrier layers were integrated with the TE legs and Cu electrodes via a one‐step SPS co‐sintering process. The output power (*P*) and conversion efficiency (*η*) were measured using a Mini‐PEM system (Advanced Riko) under vacuum, where *η* = *P*/(*P*+*Q*) = *P*/*Q*_in_ (Q is measured by the mini‐PEM). The theoretical efficiency and internal field distributions were simulated using COMSOL Multiphysics, with boundary conditions detailed in Note S6.

## 3 Results and Discussion

### 3.1 Thermodynamic Phase Evolution and Multiscale Structural Engineering

**FIGURE 2:**
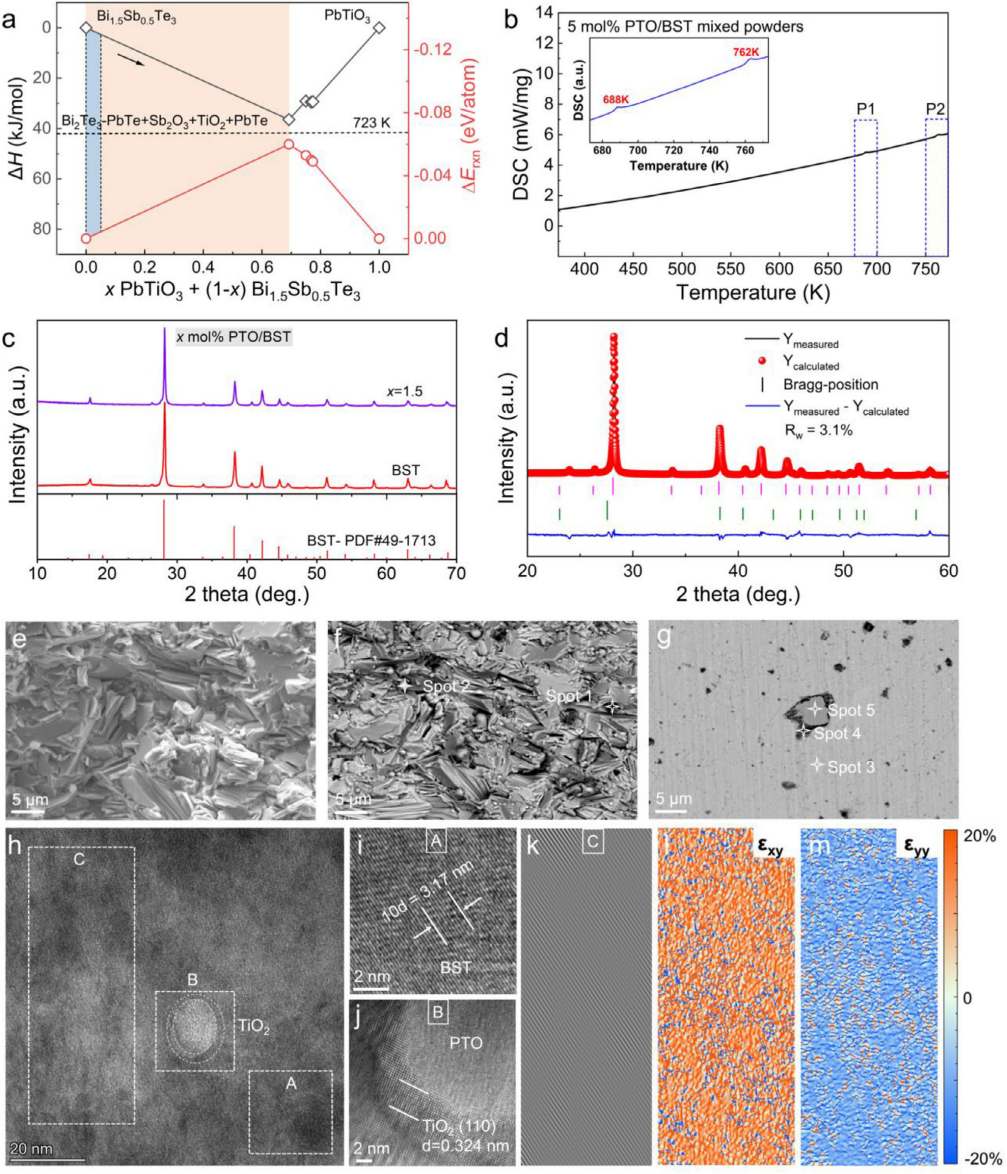
Thermodynamic stability and microstructural characterization of the PTO/BST composite. (a), Calculated activation enthalpy (Δ*H*) and reaction energy (Δ*E*) for the solid‐state reaction between PTO and BST at 723 K, derived from DFT calculations. (b), DSC profiles of the PTO/BST powder mixtures. The inset displays a magnified view of the thermal signal near the sintering temperature (∼723 K). (c), Powder X‐ray diffraction (XRD) patterns obtained for the bulk BST matrix and the composite samples with varying PTO contents. (d), Rietveld refinement of the XRD pattern for the PTO/BST composite. The experimental data (black line), calculated profile (red dots), and difference curve (bottom blue line) are shown, with vertical tick marks indicating Bragg positions. (e), Secondary electron (SE) scanning electron microscopy (SEM) image. (f), BSE image of the fractured cross‐section. (g), Backscattered electron (BSE) image of the polished surface of the PTO/BST sample (*x* = 1.5 mol%). h, Bright‐field transmission electron microscopy (TEM) image of the bulk composite. (i,j), HR‐TEM images corresponding to the regions labeled A and B in (h,k), Inverse fast Fourier transform (IFFT) image derived from the selected area C. (l,m), Geometric phase analysis (GPA) strain maps showing the lattice strain distribution of *ε*_xy_ and *ε*_yy_, respectively.

To rationalize the design of PTO/BST composites, we evaluated the energetics of the solid‐state reaction using density functional theory (DFT) calculations [34]. As illustrated in Figure 2a, the calculated reaction energy (Δ*E*_rxn_) reaches a minimum of ‐0.06 eV/atom at 723 K, indicating a strong thermodynamic driving force for the reaction between PTO and the BST matrix to form thermodynamically stable products (e.g., PbTe‐based solid solutions and oxide precipitates). Guided by these predictions, we experimentally validated the reaction kinetics via differential scanning calorimetry (DSC) (Figure 2b). The sintering temperature was rigorously selected at 723 K, which was positioned between the reaction onset (∼688 K) and the Curie transition of residual PTO (∼762 K) [35] to ensure sufficient reaction kinetics while preserving structural integrity. Structural characterization via X‐ray diffraction (XRD) and Rietveld refinement (Figure 2c,d; Figure S1 and Table S1) quantitatively corroborates this phase evolution. The Whole Pattern Fitting (WPF) analysis (*R*_wp_ ≈ 7.28%) reveals that the matrix retains its rhombohedral structure, while detecting a refined mass fraction of approximately 14.9% for the PbTe‐based phase, confirming the successful introduction of secondary phases. Compared to conventional ex situ mechanical mixing, this in situ approach ensures atomically clean interfaces and superior doping homogeneity, which are critical for maintaining high carrier mobility while maximizing phonon impedance.

To visualize the spatial distribution of these reaction products, we employed multiscale microscopic characterization. Backscattered electron (BSE) imaging and elemental mapping (Figure 2e–g; Figure S2) reveal a unique multiphase architecture: unreacted PTO cores are encapsulated by TiO_2_‐rich shells, uniformly dispersed within the BST matrix. We attribute this core–shell morphology to a diffusion‐limited mechanism where Pb atoms preferentially diffuse into the matrix (doping), while Ti‐rich species segregate at grain boundaries to form a diffusion barrier. This core‐shell morphology is attributed to the high diffusion rate of Pb into the matrix, whereas the low solubility of Ti triggers its segregation at grain boundaries to form a TiO_2_ diffusion barrier. Atomic‐scale insights from transmission electron microscopy (TEM) (Figure 2h–j) further identify the semi‐coherent hetero‐interfaces between the BST substrate and the TiO_2_ diffusion layer. Crucially, the lattice mismatch at these interfaces induces substantial lattice distortion [33]. Geometric phase analysis (GPA) of the inverse fast Fourier transform (IFFT) images visualizes intense strain fields (*ε_xy_* and *ε_yy_*) and dense incoherent dislocation arrays (Figure 2k–m). These multiscale defects, ranging from mesoscopic core–shell precipitates to atomic‐scale lattice strains, construct a hierarchical phonon scattering landscape, fundamental to the thermal transport properties discussed below.

### 3.2 Atomic Disorder Induced Electronic Optimization

**FIGURE 3:**
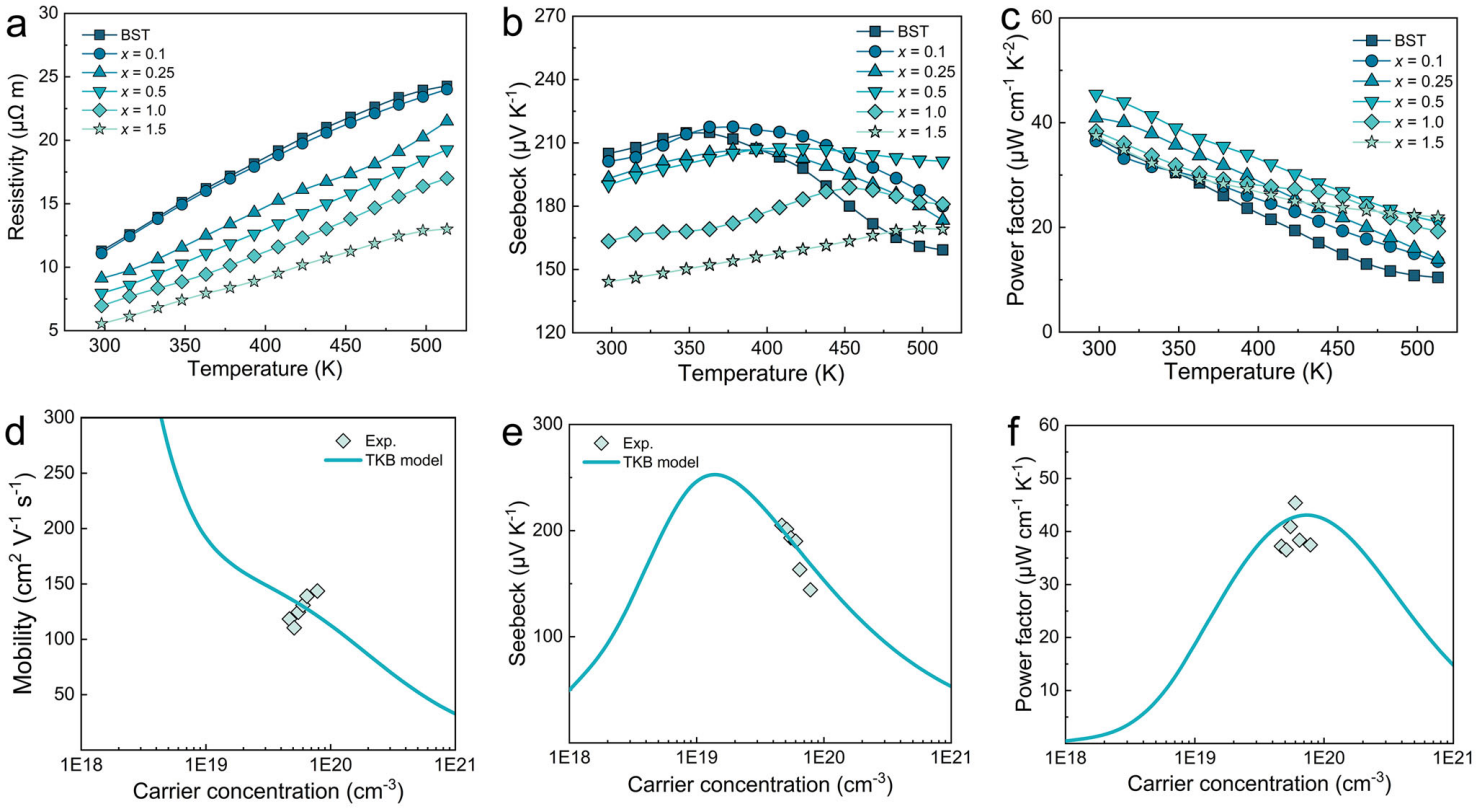
Temperature‐dependent electrical transport properties. (a–c), Temperature dependence of electrical resistivity (a), Seebeck coefficient (b), and power factor (c) for the bulk samples. (d–f), Room‐temperature measurements of electrical properties: Hall carrier concentration vs. mobility (d), Seebeck coefficient (e), and power factor (f) plotted as a function of carrier concentration. The dashed line in e indicates the Pisarenko relation.

The introduction of PTO triggers a solid‐state reaction that effectively tunes the carrier transport characteristics of the BST matrix. Hall measurement results (Figure S3a) reveal that the carrier concentration (*n*) increases monotonically with PTO content, while carrier mobility (*μ*) remains largely preserved. This behavior suggests that Pb atoms successfully occupy Sb sites to act as acceptors, providing effective hole doping without introducing severe carrier scattering centers. Consequently, the electrical resistivity decreases significantly across the entire temperature range (Figure 3a), exhibiting typical degenerate semiconductor behavior.

The temperature‐dependent Seebeck coefficient provides further insight into the electronic structure (Figure 3b). At lower temperatures (<363 K), the magnitude of *S* decreases with increasing PTO content, following the classical inverse relationship with carrier concentration described by the Pisarenko relation [36] (Figure 3e). Crucially, however, at elevated temperatures where pristine narrow‐gap BST typically suffers from severe bipolar effects (intrinsic excitation), the PTO‐doped samples exhibit a flattened Seebeck curve. This indicates that the optimized high hole concentration pushes the Fermi level deep into the valence band (consistent with DFT results), thereby effectively suppressing the thermal excitation of minority carriers (electrons).

This decoupling of transport parameters culminates in a substantial enhancement in the power factor. As shown in Figure 3c, the optimized carrier concentration balances the trade‐off between electrical conductivity and the Seebeck coefficient. The 0.5 mol% PTO/BST sample yields a maximum *power factor* of 4134 µWm^−1^K^−2^ at 333 K, representing a ∼38% enhancement over the pristine matrix. To validate the physical origin of this improvement, we modelled the transport data using the two Kane band (*E*_C_+*E*_V_) model (Figure 3d–f; Figure S3b, Note S1, and Table S2). The analysis reveals that the effective mass (m^*^) remains virtually unchanged despite the formation of the core‐shell microstructure. The preservation of mobility is largely due to the semi‐coherent nature of the in situ formed interfaces, which provides a high‐transparency pathway for charge carriers while selectively blocking phonons. This confirms that the performance boost stems primarily from the precise tuning of the E_F_ to the optimal range for power generation, rather than a fundamental alteration of the host band curvature.

### 3.3 Microscopic Insights into Atomic Substitution and Bonding

**FIGURE 4:**
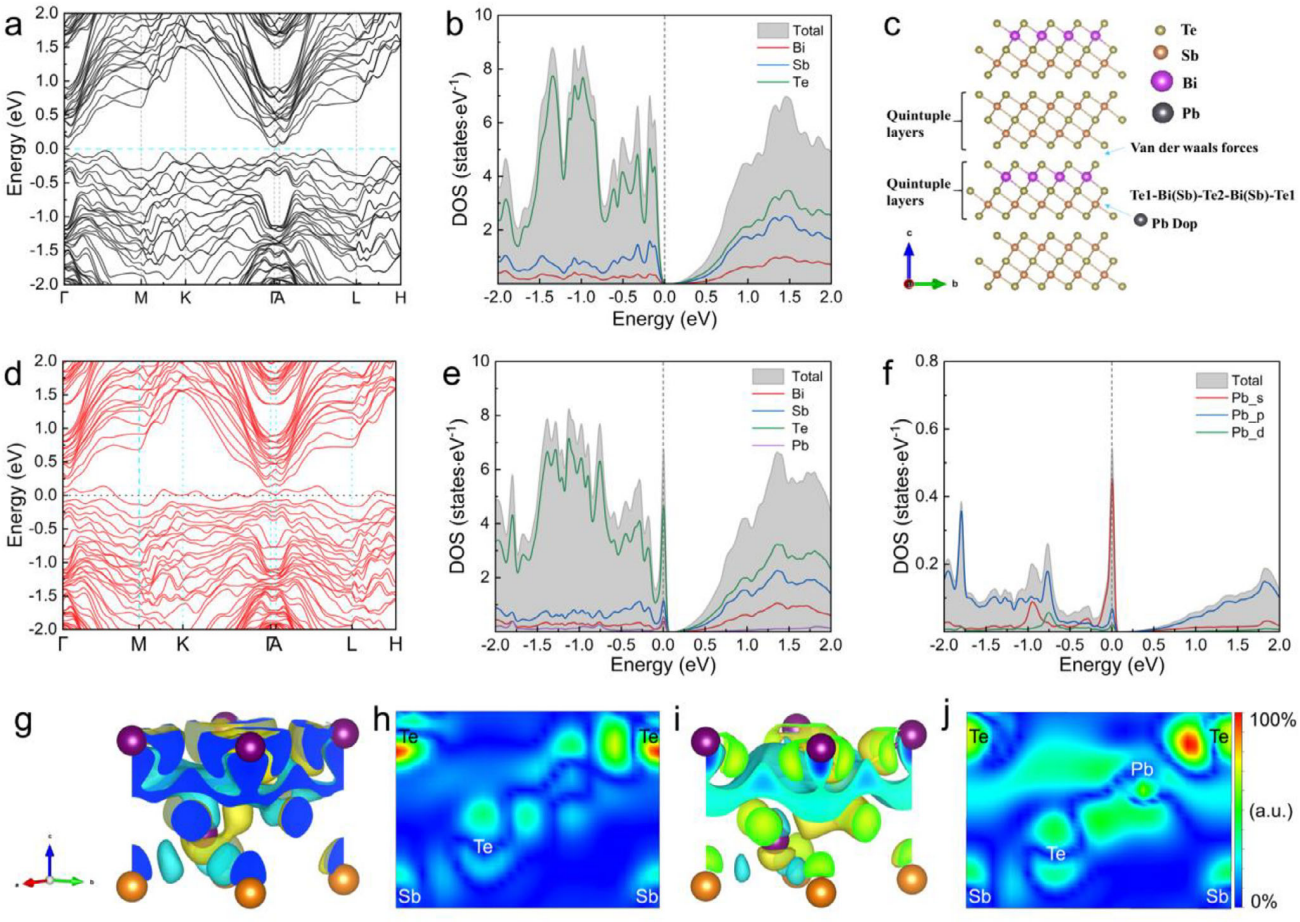
Electronic structure calculations and charge density analysis. (a,b), Electronic band structure along high‐symmetry directions (a) and TDOS (b) calculated for pristine BST. (c), Optimized supercell structure model of Pb‐doped BST used for DFT calculations. (d–f), Electronic properties of the Pb‐doped system: band structure (d), TDOS (e), and PDOS projected onto Pb orbitals (f). The horizontal dashed lines in (a, b, d, e, and f) denote the *E*_F_. (g–j), Differential charge density distributions for pristine BST (g,h) and Pb‐doped BST (i,j). The yellow and blue isosurfaces represent electron accumulation and depletion, respectively.

To elucidate the influence of Pb doping on the thermoelectric performance of the BST matrix, we performed DFT calculations [37] (see Note S6). The baseline electronic band structure and total density of states (TDOS) of pristine BST (Figure 4a,b) confirm its narrow bandgap and complex multiband features [38]. The narrow bandgap poses an intrinsic challenge, as it favors bipolar diffusion at elevated temperatures, which severely degrades the Seebeck coefficient.

Upon Pb incorporation via the solid‐state reaction method, the electronic structure undergoes a favorable reconstruction. Analysis of the Pb‐doped structure model (Figure 4c) shows that the *E*_F_ is successfully shifted deep into the valence band (Figure 4d,e), confirming the introduction of p‐type carriers and effective hole doping. This *E*_F_ shift intrinsically suppresses the detrimental bipolar effect across the measured temperature range. DFT analysis (Figure 4) confirms that Pb doping intrinsically enlarges the energy barrier for minority carrier excitation, thereby suppressing bipolar diffusion across the measured temperature range. Furthermore, the conduction band minimum (CBM) exhibits a subtle shift from the Γ point to the K/Γ path, contributing to a higher valley degeneracy. While the TDOS and projected density of states (PDOS) reveal that the *s*‐orbitals of the incorporated Pb atoms contribute significantly to the states near *E*_F_ (Figure 4e,f).

**FIGURE 5:**
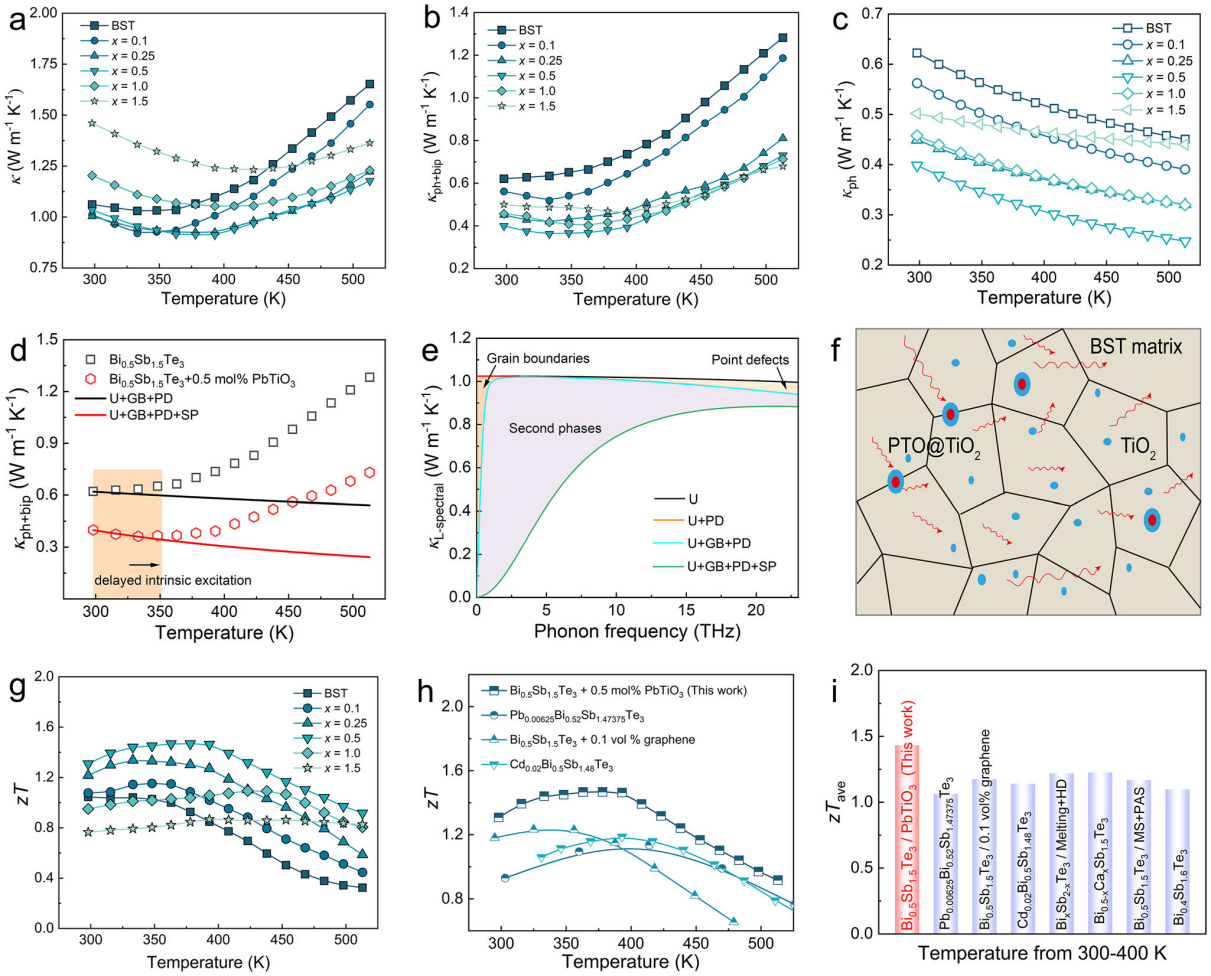
Thermal transport properties and thermoelectric performance evaluation. (a–c), Temperature dependence of total thermal conductivity (tot) (a), combined lattice and bipolar thermal conductivity (*κ*_ph_+*κ*_bi_) (b), and *κ*_ph_ (c) for bulk samples with varying PTO contents. (d), Experimental (points) and calculated (lines) lattice thermal conductivity based on the Debye‐Callaway model, showing the dominant role of interface scattering in reducing *κ*_ph_. (e), Spectral lattice thermal conductivity derived for different phonon scattering mechanisms. The curves illustrate the cumulative reduction in conductivity attributed to specific scattering sources. (f), Schematic illustration of the multiscale phonon scattering centers within the PTO/BST composites. (g), *zT* of the bulk samples. (h), Comparison of thermoelectric performance optimization strategies for BST reported in recent literature. (i), Comparison of *zT*_ave_ values in the temperature range of 300–400 K for the PTO/BST sample and other state‐of‐the‐art BST‐based systems.

We further investigated the chemical bonding via differential charge density analysis (Figure 4g–j). In contrast to the relatively uniform charge distribution of undoped BST, Pb incorporation leads to a marked enhancement in localized electronic density around the dopant site. This localization signifies an optimized chemical environment and confers stronger chemical bonding, which is conducive to stabilizing the hole population and minimizing phonon scattering at the atomic level. Collectively, the DFT analysis validates that the solid‐state reaction strategy is highly effective in optimizing the carrier concentration and intrinsically mitigating thermal degradation by suppressing bipolar effects, thus underpinning the superior thermoelectric performance observed in Figures 3 and 5. Lead typically exhibits a positive valence state because of its tendency to lose outer‐shell electrons to achieve a stable configuration. Our Hall effect measurements (Figure S3) shows that the hole concentration increases monotonically with PTO addition. Since Ti typically exhibits a 3+ or 4+ state and does not act as an electron acceptor here, we conclude that Pb^2+^ substitutes for Bi^3+^ or Sb^3+^, thereby accepting electrons from the matrix. Therefore, Pb is considered to occupy the cation site in the structural models for DFT calculation.

### 3.4 Thermal Transport Mechanism and Thermoelectric Performance

Thermal management in narrow‐gap BST semiconductors is critical due to the detrimental contribution of bipolar diffusion (*κ*_bip_) and *κ*_e_ [39] (Figure S4) to the total thermal conductivity. As shown in Figure 5a–c, the PTO/BST composites exhibit a marked suppression in *κ* and *κ*_ph_ compared to the pristine matrix. This reduction originates from a synergistic mechanism: the in situ formed core‐shell microstructures and point defects introduce dense scattering centers, while the optimized carrier concentration mitigates the bipolar effect by suppressing minority carrier excitation at elevated temperatures.

To quantitatively decouple the phonon scattering mechanisms, we fitted the *κ*_ph_ data using the Debye‐Callaway model (Figure 5d; Notes S3, S4, and Table S3). The spectral lattice thermal conductivity analysis (Figure 5e) reveals that the scattering from second‐phase interfaces (SP) dominates the phonon impedance. The nanoscale PTO@TiO_2_ interfaces effectively scatter mid‐ to low‐frequency phonons, which typically possess long mean free paths and escape scattering by point defects (Figure 5f). Specifically, the hierarchical core‐shell interfaces and associated strain fields (Figure 2k–m) act as a frequency‐selective barrier, predominantly scattering mid‐to‐low frequency phonons that escape scattering by point defects. Statistical image analysis (Figure S5) reveals a mean nanoparticle size of ∼ 82 nm. The 0.5 mol% PTO composition provides an optimal spatial density for maximizing interfacial phonon scattering; beyond this threshold (e.g., ≥1.0 mol%), the aggregation of secondary phases compromises the thermal blocking effect due to their intrinsically higher thermal conductivity. However, a trade‐off is observed at higher PTO contents (*x* ≥1.5 mol%); the presence of excessive PTO and TiO_2_ phases characterized by high intrinsic thermal conductivities (∼4 and ∼8.5 Wm^−1^K^−1^, respectively) [40, 41] partially counteracts the interface scattering effect, leading to a slight upturn in *κ*_ph_.

The simultaneous optimization of electrical transport and thermal insulation translates into a significant enhancement in the figure of merit. The 0.5 mol% PTO/BST composite achieves a peak *zT* of 1.47 at 333 K, representing a ∼36% improvement over pristine BST (Figure 5g). Importantly, the performance enhancement is broad: the average *zT*_ave_ reaches 1.43 within the critical near‐room‐temperature range of 300–400 K (Figure 5i). It is worth noting that conventional pure Pb doping strategies primarily optimize the electronic structure but lack mesoscale phonon barriers (Figure S6). The significantly higher *zT*_ave_ achieved in this work unambiguously isolates the specific advantage of our in situ approach: the simultaneous generation of PTO@TiO_2_ core‐shell architectures that provide an additional ∼ 36% reduction in lattice thermal conductivity without compromising hole mobility. As benchmarked in Figure 5h, this performance surpasses that of recent BST‐based materials modified via single‐phase addition or isolated doping [15, 16, 42], highlighting the superiority of our in situ reaction strategy in decoupling electron and phonon transport for high‐efficiency thermal energy harvesting.

### 3.5 Thermoelectric Module Design and Power Generation Performance

**FIGURE 6:**
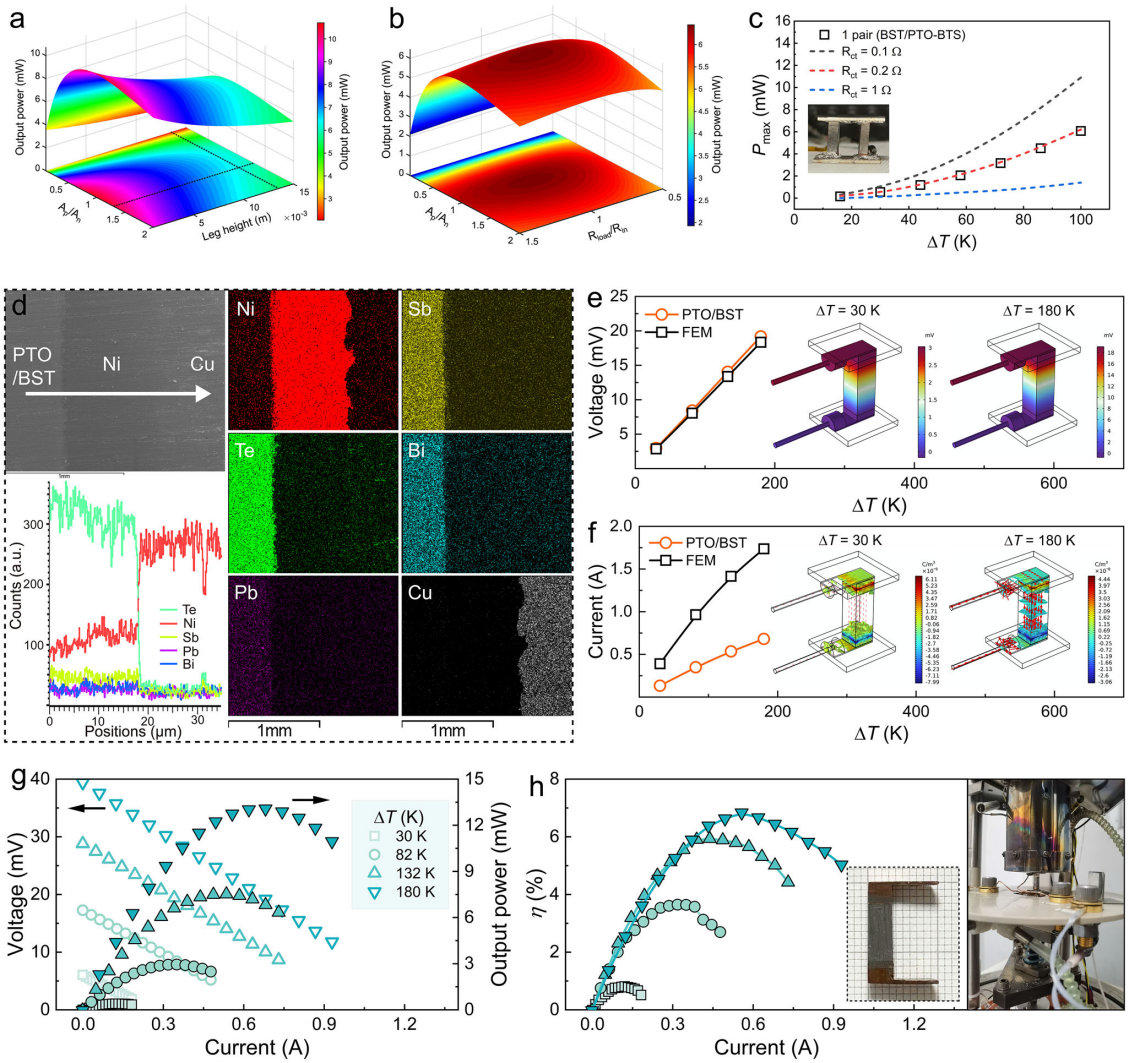
Design optimization, interfacial characterization, and power generation performance of the TE module. (a,b), Theoretical optimization of the maximum output power (*P*_max_) as a function of thermoelectric leg height (*l*) and cross‐sectional area ratio (*A*_p_/*A*_n_) (a), and as a function of the external‐to‐internal resistance ratio (*R*_load_/*R*_in_) and area ratio (b), calculated at a Δ*T* of 100 K. (c), Output power of a single unicouple consisting of p‐type PTO/BST and n‐type Bi_2_Te_2.7_Se_0.3_ legs under various Δ*T*. (d), Cross‐sectional SEM image, EDS elemental mapping, and EDS line scan profiles (indicated by the arrow) across the hot‐side electrode/thermoelectric leg interface. (e,f), Experimental output voltage (e) and current (f) of the fabricated module. The insets display the corresponding 3D FEM simulations of electric potential and current density distributions. (g,h), Output characteristics of the as‐prepared module under different Δ*T*: output voltage and power vs. current (g), and *η* vs. current (h). The right‐side panels show optical photographs of the assembled module and the measurement setup (Mini‐PEM).

To maximize the output power density, we rationalized the geometric design of the thermoelectric (TE) module to accommodate the intrinsic transport asymmetry between the p‐type PTO/BST and n‐type Bi_2_Te_2.7_Se_0.3_ legs (Figure S7 and Note S5). As indicated by the theoretical calculations in Figure 6a,b, to match the impedance of the external load, we optimized the geometric factor (*A*/*l*). Guided by this optimization, we fabricated a single‐pair module (unicouple) with a leg height of 13.5 mm and a cross‐sectional area ratio of A_p_/A_n_  ≈ 1.3.

A critical challenge in realizing high‐performance devices is minimizing the contact resistance (*R*_ct_) at the electrode/TE leg interface. Our simulations reveal that elevated *R*_ct_ significantly suppresses power output, particularly in modules with high‐aspect‐ratio legs (Figure S8). To overcome this bottleneck, we employed a one‐step SPS technique to co‐press the Cu electrodes, Ni diffusion barriers, and TE legs. The resulting interface exhibits a robust metallurgical bond with no visible delamination (Figure 6d). Elemental mapping and line scans confirm that the Ni layer effectively inhibits chemical interdiffusion between Cu and the TE matrix, ensuring low interfacial resistance and long‐term stability. The Ni barrier is indispensable for maintaining module efficiency, as it prevents the rapid diffusion of Cu into the BST matrix, which would otherwise lead to severe performance degradation.

The experimental performance of the assembled module shows agreement with 3D finite‐element method (FEM) simulations (Figure 6e,f; Figure S9, Note S6, and Table S4). The measured output voltage and current increase linearly with the temperature difference, corroborating the ohmic contact behavior (Figure 6g). The internal resistance derived from the *V*–*I* slope matches the theoretical design (Figure S10a). Consequently, the module delivers a maximum output power of 13.1 mW at a Δ*T* of 180 K, corresponding to a conversion efficiency of ∼7% (Figure 6h; Figure S10b–d), demonstrating the potential of PTO/BST composites for practical waste heat recovery.

## 4 Conclusion

In summary, we have demonstrated a robust strategy that synergistically manipulates atomic disorder and mesoscale architectures in BST‐based thermoelectrics. The beauty of this approach lies in its multiscale mechanism: atomic‐scale Pb substitution optimizes the Fermi level to intrinsically suppress bipolar diffusion at elevated temperatures, while mesoscale core–shell PTO@TiO_2_ precipitates create a frequency‐selective barrier that effectively scatters mid‐ to low‐frequency phonons. This in situ reaction‐driven decoupling of electron and phonon transport yields a peak *zT* of 1.47 at 333 K, overcoming the performance bottleneck of conventional near‐room‐temperature materials. Crucially, we bridged the gap between fundamental material science and practical application through holistic device engineering. By integrating geometric optimization with a one‐step sintering technique that minimizes interfacial contact resistance, we successfully translated the high intrinsic *zT* into a module‐level conversion efficiency of ∼7% and an output power of 13.1 mW under a temperature difference of 180 K. This work not only provides a commercially viable solution for low‐grade heat recovery but also establishes a generalizable protocol for designing high‐performance functional materials via defect engineering and microstructure modulation. Looking forward, this in situ reaction‐driven paradigm could be readily extended to other complex material systems such as half‐Heuslers or skutterudites, where the decoupling of interdependent transport properties remains a grand challenge. The criteria for selecting precursors in other systems, such as SnSe and Mg_3_Sb_2_, include strong in situ thermodynamic driving forces and the ability to release species that facilitate simultaneous doping and secondary‐phase segregation.

## Author Contributions

Jiwu Xin, Ting Zhang, and Qinghui Jiang conceived and designed the study. Bo Wang, Qinghui Jiang, Jiwu Xin, and Jinfeng Dong carried out the experiments and performed the material characterizations. Jiwu Xin, Jinfeng Dong, and Chengyun Xu conducted module fabrication and performance measurements. The manuscript was written, edited, and revised by Jiwu Xin, Jinfeng Dong, Lei Wei, Ting Zhang, and Qinghui Jiang. All authors contributed to the discussion and interpretation of the results. This work was supported by the National Natural Science Foundation of China (52273293, 52172187, 51772019, and 51572098), the National Key Research and Development Program of China (2023YFB3809800), the Nanjing International Science and Technology Cooperation Project (202512034), and the Royal Society (IEC/NSFC/170290).

## Conflicts of Interest

The authors declare no conflicts of interest.

## Supporting information




**Supporting File**: advs74899‐sup‐0001‐SuppMat.docx.

## Data Availability

The data that support the findings of this study are available from the corresponding author upon reasonable request.
