# Supplementary material for: Manipulating Atomic Disorder and Mesoscale Architectures for High‐Efficiency Thermoelectric Modules
Source: Adv Sci (Weinh). 2026 May 7;13(33):e74899. doi: 10.1002/advs.74899 (PMC13271610; doi:10.1002/advs.74899)
Supplement: Supplementary file 1 — Supporting File: advs74899‐sup‐0001‐SuppMat.docx. [file ADVS-13-e74899-s001.docx]

# Supplementary Information

**Manipulating atomic disorder and mesoscale architectures for high-efficiency thermoelectric modules**

Jiwu Xin^1,2,3^, Bo Wang^1^, Chengyun Xu^2^, Wang Li^1^, Pengyu Zhang^4^, Dongwang Yang^5^, Yongke Wang^6^, Jinfeng Dong^5,^*, Lei Wei^2,*^ , Ting Zhang^4,7,*^, Qinghui Jiang^1,8,*^

^1^ State Key Laboratory of Materials Processing and Die and Mould Technology, Huazhong University of Science and Technology, Wuhan 430074, PR China

^2^ School of Electrical and Electronic Engineering, Nanyang Technological University, 50 Nanyang Avenue, Singapore 639798, Singapore

^3^ Hangzhou International Innovation Institute, Beihang University, 311115, Hangzhou, China

^4^ Institute of Engineering Thermophysics, Chinese Academy of Sciences, Beijing 100190, China

^5^ School of Materials Science and Engineering, Nanyang Technological University, Singapore 639798, Singapore

^6^  Engineering Department, University of Miami, 4808S CONWAY RD ORLANDO FL 32812, USA

^7^  University of Chinese Academy of Sciences, Nanjing 211135, China

^8^  School of Microelectronics, Wuhan Textile University, Wuhan, 430077, China

* Corresponding author: jinfeng.dong@ntu.edu.sg; wei.lei@ntu.edu.sg; zhangting@iet.cn; qhjiang@hust.edu.cn

## Supplementary Notes

**Supplementary Note 1. Calculation of Kane energy band (KEB) model**

In the two-band Kane model incorporating contributions from both conduction band (CB) and valence band (VB), the total thermoelectric transport properties are derived as follows. The subscript C denotes conduction band–related quantities, while V denotes valence band–related counterparts.

**Conduction Band Contributions:**

- Hall carrier concentration

$p_{C}=\frac{N_{C}\left( 2m_{b}^{*}k_{B}T \right)^{\frac{3}{2}}F_{3/2,0}^{0}\left( , \right)}{3A_{C}{}^{2}\hbar^{3}}$ (S1)

- Seebeck coefficient

$S_{C}=\frac{k_{B}}{e}\left[ \frac{F_{1,-2}^{1}\left( , \right)}{F_{1,-2}^{0}\left( , \right)}- \right]$ (S2)

- Hall carrier mobility

${}_{C}=\frac{2A_{C}\hbar^{4}eC_{l}}{m_{I}^{*}\left( 2m_{b}^{*}k_{B}T \right)^{\frac{3}{2}}E_{C,def}^{2}}\frac{3F_{1,-2}^{0}\left( , \right)}{F_{\frac{3}{2,0}}^{0}\left( , \right)}$ (S3)

- Hall factor

$A_{C}=\frac{3K_{C}(K_{C}+2)}{{(2K_{C}+1)}^{2}}\frac{F_{1/2,-4}^{0}F_{3/2,0}^{0}}{\left( F_{1,2}^{0} \right)^{2}}$ (S4)

- Electrical conductivity

${}_{C}=p_{C}{}_{C}e=\frac{2{\hbar e}^{2}{N_{C}C}_{l}}{m_{I}^{*}E_{C, def}^{2}}F_{1,2}^{0}\left( , \right)$ (S5)

- Electronic Thermal Conductivity (via Wiedemann–Franz Law):

${}_{C, e}=L_{C}{}_{C}T$ (S6)

- Lorenz number:

$L_{C}=\left( \frac{k_{B}}{e} \right)^{2}\left[ \frac{F_{1,-2}^{2}\left( , \right)}{F_{1,-2}^{0}\left( , \right)}-\left( \frac{F_{1,-2}^{1}\left( , \right)}{F_{1,-2}^{0}\left( , \right)} \right)^{2} \right]$ (S7)

- Generalized Fermi integration:

$F_{m,k}^{n}\left( , \right)=\int_{0} \left[ -\frac{f()}{} \right]{}^{n}{(+{}^{2})}^{m}\left[ {(1+2)}^{2}+2 \right]^{\frac{k}{2}}d$ (S8)

Where *η* is the reduced Fermi level, *β* = *K_B_T*/*E_g_* is the reciprocal reduced band gap, *K_B_*, *ħ*, *N*, *C_l_* are the Boltzmann constant, reduced Planck constant, band degeneracy and the combination of elastic constants, respectively. *E_def_* is the deformation potential and $m_{I}^{*}$ is the inertial effective mass.

**Total electrical transport properties:**

- Total Hall coefficient:

$R_{\mathrm{Htot}}=\frac{R_{\mathrm{HC}}{{}_{C}}^{2}+R_{\mathrm{HV}}{{}_{V}}^{2}}{{({}_{C}+{}_{V})}^{2}}$ (S9)

- Total Hall carrier concentration:

$n_{\mathrm{Htot}}=\frac{1}{eR_{\mathrm{Htot}}}$ (S10)

- Total electrical conductivity:

${}_{\mathrm{tot}}={}_{C}+{}_{V}$ (S11)

- Total Hall carrier mobility:

${}_{\mathrm{Htot}}={}_{\mathrm{tot}}R_{\mathrm{Htot}}$ (S12)

- Total Seebeck coefficient:

$S_{\mathrm{tot}}=\frac{S_{C}{}_{C}+S_{V}{}_{V}}{{}_{C}+{}_{V}}$ (S13)

- Total Lorenz number:

$L_{\mathrm{tot}}=\frac{L_{C}{}_{V}+L_{C}{}_{V}}{{}_{C}+{}_{V}}$ (S14)

- *zT* based on KEB model:

${zT}_{\mathrm{KEB}}=\frac{{}_{\mathrm{tot}}S_{\mathrm{tot}}^{2}T}{{}_{L, ave}+{}_{e}}$ (S15)

All relevant material parameters for the above calculations are provided in Supplementary Table S2.

**Supplementary Note 2. Details of heat capacity calculations**

The total specific heat capacity *C*_p,tot_ consists of two components: the phonon contribution *C*_p,ph_ and the lattice dilation contribution *C*_p,D_. The temperature-dependent expression for *C*_p,tot_ is given by^[1]^:

$C_{p,tot}=C_{p,ph}\left( T \right)+C_{p,D}\left( T \right)$ (S16)

**Phonon Contribution (Debye Model)**

The phonon-specific heat *C*_p,ph_ is modeled using the Debye approximation:

$C_{p,ph}\left( \frac{T}{{}_{D}} \right)=9R\left( \frac{T}{{}_{D}} \right)^{3}\int_{0}^{\frac{{}_{D}}{T}} \frac{x^{4}e^{x}}{\left( e^{x}-1 \right)^{2}}dx$ (S17)

Where, *θ*_D_ is the Debye temperature, *x* = ħ*ω*/*k*_B_*T* (with ω denoting the phonon frequency) as the reduced phonon frequency, ħ is the reduced Planck constant, *k*_B_ is the Boltzmann constant.

#### **Lattice Dilation Contribution**

The dilation component *C*_p,D_ includes both electronic and phonon dilation contributions:

$C_{p,D}=C_{ele,D}\left( T \right)+C_{ph,D}\left( T \right)=\frac{9BT{}^{2}}{{d10}^{6}}$ (S18)

Where *B* is the isothermal bulk modulus, *α* is the linear coefficient of thermal expansion, and *d* is the sample density. Given that the dilation effect predominantly arises from acoustic phonons and that the phonon contribution *C*_p,ph_ significantly exceeds the electronic contribution *C*_p,ele_, the electronic component can be neglected in most practical calculations.

**Supplementary Note 3. Prediction of alloyed lattice thermal conductivity using the Callaway model**

According to Callaway’s theory, the lattice thermal conductivity of a defect-containing material can be expressed as^[2,3]^:

$\frac{\kappa_{\mathrm{lat}}}{\kappa_{lat,p}}=\frac{\arctan\mu}{\mu}$ (S19)

Where *κ*_lat_ and *κ*_lat,p_ are the lattice thermal conductivity of the alloyed or defect-containing system, and lattice thermal conductivity of the pristine (parent) material, respectively. *μ* is a dimensionless scattering parameter, defined as^[4]^:

$\mu=\left( \frac{\pi^{2}\theta_{D}V}{{hv}^{2}}\kappa_{lat,p}Г \right)^{\frac{1}{2}}$ (S20)

Here, *V* and h are the average atomic volume and Planck constant, respectively. The Debye temperature *θ*_D_ is given by^[5]^:

${}_{D}=\frac{hv}{k_{B}}\left( \frac{3}{4V} \right)^{\frac{1}{3}}$ (S21)

The average phonon velocity 𝑣 is the average speed of a phonon, calculated using *v* = 3^1/3^(*v*_L_^-3^+2*v*_T_^-3^)^-1/3^ (with v_T_ and v_L_ respectively denoting the transverse and longitudinal sound velocities).

**Supplementary Note 4. Callaway model incorporating multiple phonon scattering mechanisms**

The lattice thermal conductivity κ_L_ can be calculated based on the Callaway model using the following integral expression^[2,6]^:

${}_{L}=\frac{k_{B}}{2{}^{2}}\left( \frac{k_{B}}{\hbar} \right)^{3}\int_{0}^{{}_{D}/T} \left( x \right)\frac{x^{4}e^{x}}{\left( e^{x}-1 \right)^{2}}$ (S22)

Here, 𝜈 is the average phonon group velocity, ${}_{D}$ is the Debye temperature, and τ(x) is the total phonon relaxation time. The integrand and prefactor together define the spectral lattice thermal conductivity (*κ*_L,spectral_), namely^[7,8]^:

${}_{L, spectral}=\frac{k_{B}}{2{}^{2}}\left( \frac{k_{B}}{\hbar} \right)^{3}\left( x \right)\frac{x^{4}e^{x}}{\left( e^{x}-1 \right)^{2}}$ (S23)

The total relaxation time 𝜏 accounts for various phonon scattering mechanisms, including:

- Umklapp scattering and normal process:

${}_{U}^{-1}=\frac{\hbar{}^{2}{}^{2}T}{M{}^{2}{}_{D}}e^{{-{}_{D}}/{3T}}$ (S24)

- Electron-phonon scattering

${}_{\mathrm{EP}}^{-1}=\frac{E_{\mathrm{def}}^{2}\left( m^{*} \right)^{2}k_{B}T}{2\hbar^{4}dv}c$ (S25)

- Point defect scattering

${}_{\mathrm{PD}}^{-1}=\frac{V}{4{}^{2}}{}^{4}$ (S26)

- Grain boundary scattering

${}_{\mathrm{GB}}^{-1}=\frac{}{L}$ (S27)

Where $M$ is the average atomic mass, *γ* is the Grüneisen parameter, *β* is the ratio between normal process and Umklapp phonon scattering, *c* is the electron-phonon scattering factor, and *L* is the grain size. All related material parameters used for the model are summarized in Supplementary Table S3.

**Sensitivity analysis of the Debye-Callaway model:**

To rigorously evaluate the robustness of our proposed frequency-selective phonon scattering mechanism and to determine the impact of parameter uncertainties, a sensitivity analysis of the Debye-Callaway model was conducted.In the model, the total lattice thermal conductivity (κ_L_) is determined by the cumulative relaxation time (*τ*_C_), which includes contributions from Umklapp scattering (*τ*_U_^-1^), grain boundary scattering (*τ*_B_^-1^), point defect scattering (*τ*_PD_^-1^), and nanoparticle/interface scattering (*τ*_NP_^-1^). While most parameters (such as sound velocity, *v*_s_, and carrier concentration, *n*) are experimentally fixed, the point defect scattering parameter (*Γ*) and the effective grain size (*L*) inherently carry certain estimation uncertainties.To conduct the sensitivity analysis, we systematically varied *Γ* and *L* by ±20% relative to their optimal fitted baseline values for the 0.5 mol% PTO/BST sample, and observed the resulting theoretical κ_L_ variations across the 300–400 K range:

**1. Sensitivity to Grain Size (*L*):** Grain boundary scattering predominantly affects ultra-low-frequency phonons. By varying *L* by ±20% (simulating the statistical variance in grain dimensions observed in SEM), the calculated total κ_L_ exhibited a negligible fluctuation of less than ±2.5%. This mathematical insensitivity confirms that microscale grain boundaries are insufficient to explain the massive ~36% reduction in κ_L_ observed experimentally.

**2. Sensitivity to Point Defect Parameter (*Γ*):** Point defects primarily scatter high-frequency phonons. To simulate a worst-case scenario where point defect scattering is severely underestimated, we artificially increased *Γ* by 20%. Although this yielded a slight further reduction in the theoretical κ_L,_ specifically at high frequencies, the model still significantly overestimated the total κ_L_ compared to the experimental data.

Crucially, if the nanoparticle scattering term (*τ*_NP_^-1^) is entirely removed from the Callaway equation, adjusting *L* and *Γ* even to extreme, physically unrealistic bounds fails to reproduce the massive κ_L_ reduction observed in the 0.5 mol% PTO/BST sample. The sensitivity analysis unequivocally demonstrates that the theoretical curve can only match the experimental data when the strong mid-to-low frequency scattering term from the PTO@TiO_2_ core-shell interfaces is included. Therefore, the interfacial scattering mechanism proposed in this work is highly robust and dictates the ultimate thermal transport behavior.

**Supplementary Note 5. Power Generation Calculation for the Thermoelectric Device**

The thermoelectric conversion efficiency 𝜂 of a device under a given temperature gradient can be expressed as:

$=\frac{\overline{S}^{2}(T_{h}-T_{c})R_{\mathrm{load}}}{\frac{1}{2}\overline{S}^{2}(T_{h}+T_{c})\overline{R}+\overline{K}\left( {\overline{R}+R}_{\mathrm{load}} \right)^{2}}$ (S28)

Where $\overline{R}$ are the sums of $\overline{R_{0}}$, $R_{\mathrm{ct}}$. $R_{\mathrm{th}}$, $\overline{R_{0}}$, $R_{\mathrm{ct}}$, $R_{\mathrm{th}}$, $\overline{S}$, and $\overline{K}$ are the resistances contributed by thermoelectric legs, the resistances from the contact layer, the interface thermal resistance, the Seebeck coefficient of the device, and the thermal conductance of the thermoelectric legs, respectively.

- Effective Seebeck Coefficient:

$\overline{S}=\frac{1}{T_{h}-T_{c}}\int_{T_{c}}^{T_{h}} \left( S_{p}(T)-S_{n}(T) \right)dT$ (S29)

- Total Leg Resistance:

$\overline{R_{0}}=\frac{H_{n}}{A_{n}}\frac{1}{T_{h}-T_{c}}\int_{T_{c}}^{T_{h}} \left( {}_{n}(T) \right)dT+\frac{H_{p}}{A_{p}}\frac{1}{T_{h}-T_{c}}\int_{T_{c}}^{T_{h}} \left( {}_{p}(T) \right)dT$ (S30)

- Thermal Interface Resistance:

$R_{\mathrm{th}}=\frac{N}{{(T}_{h}-T_{c})({CTE}_{c}+{CTE}_{h})A_{\mathrm{np}}}\left[ \int_{T_{c}}^{T_{h}} \left( S_{p}(T)-S_{n}(T) \right)dT \right]^{2}$ (S31)

- Effective Thermal Conductance:

$\overline{K}=\frac{A_{n}}{H_{n}}\frac{1}{T_{h}-T_{c}}\int_{T_{c}}^{T_{h}} \left( K_{n}(T) \right)dT+\frac{A_{p}}{H_{p}}\frac{1}{T_{h}-T_{c}}\int_{T_{c}}^{T_{h}} \left( K_{p}(T) \right)dT$ (S32)

Where 𝑁, *H*_n_, *H*_p_, 𝜌_n𝑐_, 𝜌_pc_, 𝐴_n_, 𝐴_p_, *T*_h_, and *T*_c_ are the number of thermocouples, the leg length, the contact resistivity, the cross-sectional area of *n*- and *p*-type legs, and the hot-side and cold-side temperatures, respectively. *CTE*_c_ and *CTE*_h_ represent the thermal contact resistivities of the cold and hot sides. All material and geometric parameters used in this model, as well as those applied in 3D-FEM simulations, are listed in Supplementary Table S4.

**Supplementary Note 6. Numerical Modelling**

The thermoelectric behavior of the device was numerically modeled by considering the coupled effects of the Seebeck effect, Peltier effect, Thomson effect, and Joule heating. The governing equations are:

$\nabla(\kappa\nabla T)+\frac{J^{2}}{\sigma}-TJ.\left[ \left( \frac{\partial S}{\partial T} \right)\nabla T+(\nabla S)_{T} \right]=0$ (S33)

$\nabla\cdot J=0$ (S34)

Where *T*, *κ*, *σ*, and *S* are represented by temperature, the thermal conductivity, electrical conductivity, and the Seebeck coefficient, respectively. The current density *J* is described by:

$J=-\sigma(\nabla V+S\nabla T)$ (S35)

Where 𝑉 is the electrostatic potential. These coupled differential equations were solved using the finite element method (FEM) via the commercial software COMSOL Multiphysics, which enables full-field simulation of temperature and voltage distributions across the thermoelectric module under various operating conditions.

## Supplementary Figures

##
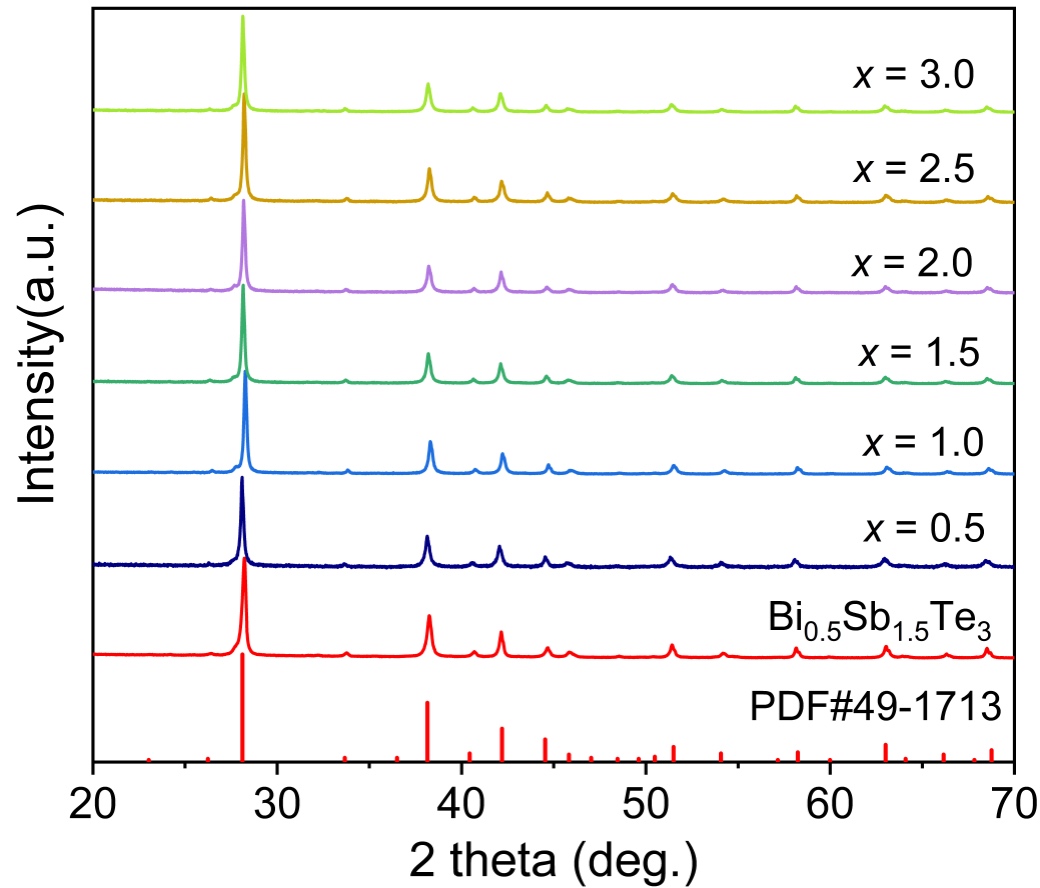


**Fig. S1** Bulk XRD patterns of bulk BST composites with varying PTO contents (*x* = 0.5–3 mol%)

##
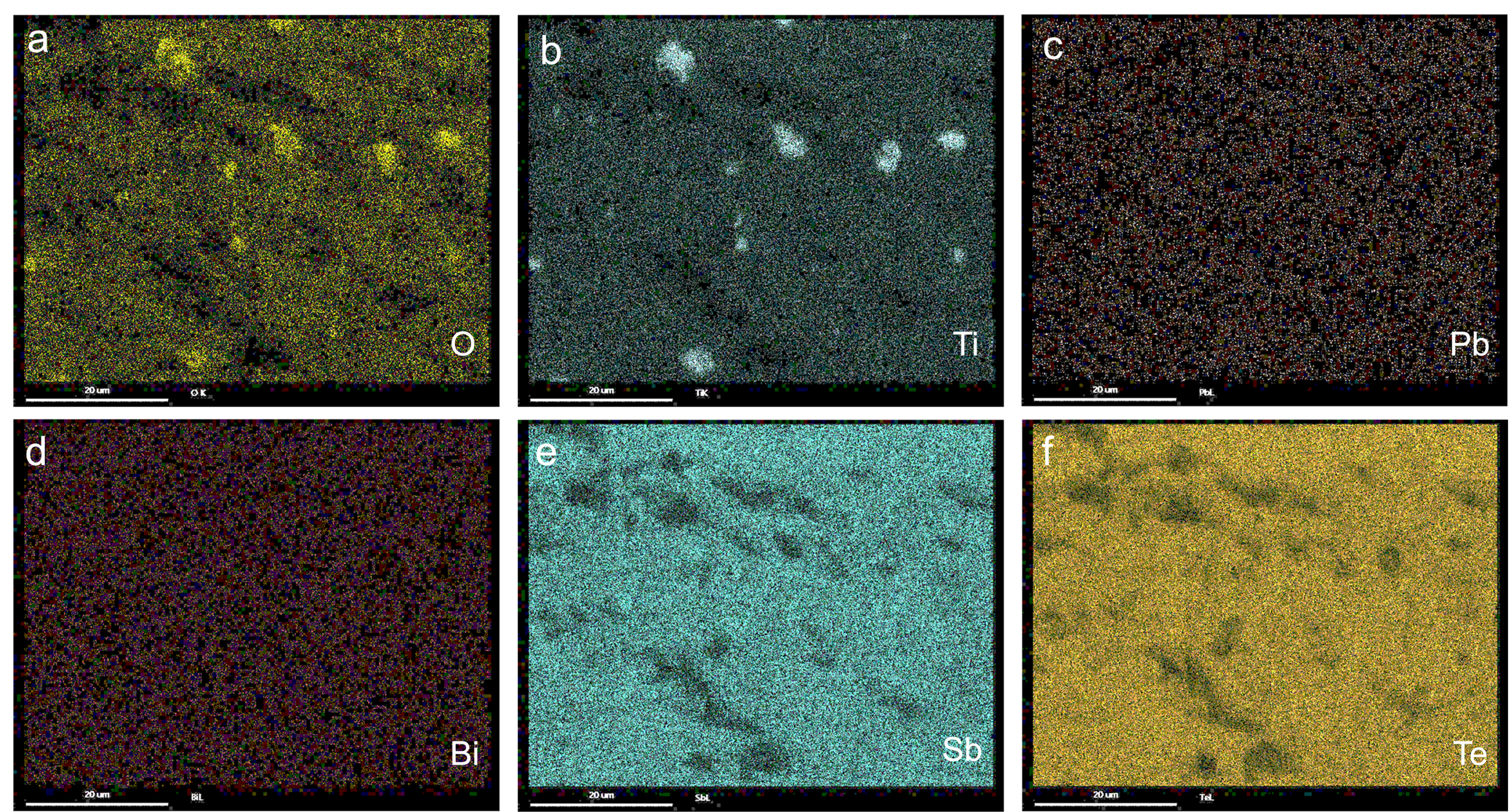


**Fig. S2 a-d,** EDS elemental mapping images for O, Ti, Pb, Bi, Sb, and Te corresponding to the microstructural area shown in **Fig. 2f**.

##
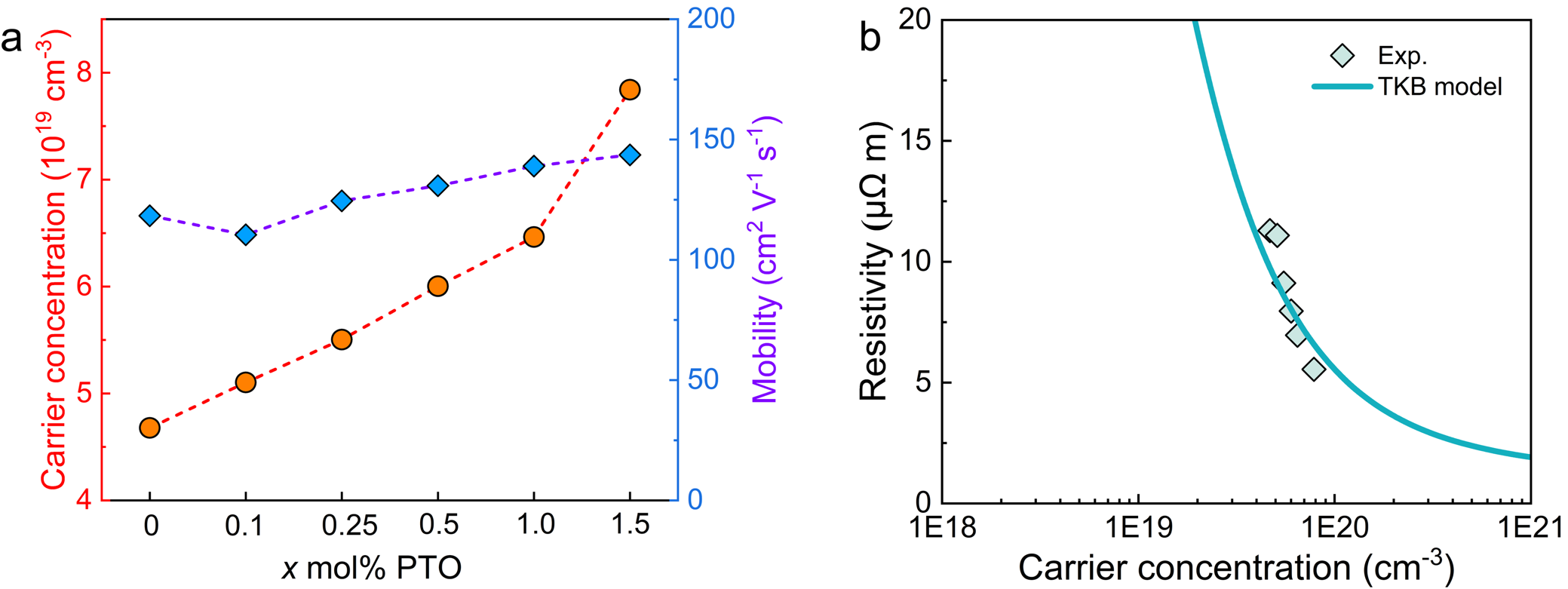


**Fig. S3 a,** Hall carrier concentration and carrier mobility of samples as a function of PTO content. **b,** Electrical resistivity plotted against carrier concentration.





**Fig. S4** **a**, Temperature dependence of the Lorenz number (*L*). **b**, Electronic thermal conductivity (*κ*_e_) as a function of temperature.





**Fig. S5** Quantitative microstructural analysis of the optimized 0.5 mol% PTO/BST sample. (**a, b**) Large-area SEM images revealing the uniform spatial distribution of the in situ generated PTO@TiO_2_ core-shell nanoparticles embedded within the matrix. Scale bars: 500 nm. (**c**) Particle size distribution histogram extracted from the corresponding SEM micrographs using ImageJ software.

**

**

**Fig. S6** Benchmarking the thermoelectric performance of the in situ generated 0.5 mol% PTO/BST composite against conventional pure Pb-doped Bi_2_Te_3_-based systems from literature^[9–11]^. Temperature-dependent (**a**) lattice thermal conductivity (*κ*_L_) and (**b**) figure of merit (*zT*).

**

**

**Fig. S7 a–d,** Temperature dependence of thermoelectric properties for n-type Bi_2_Te_2.7_Se_0.3_: electrical conductivity (*σ*) (**a**), Seebeck coefficient (*S*) (**b**), total thermal conductivity (*κ*_tot_) (**c**), and figure of merit (*zT*) (**d**).

**

**

**Fig. S8 a,** Calculated output power (*P*_out_) as a function of contact resistance (*R*_c_) for different thermoelectric leg heights (*l*). **b,** Relative reduction in output power (Δ*P/P*) versus contact resistance at various leg heights.


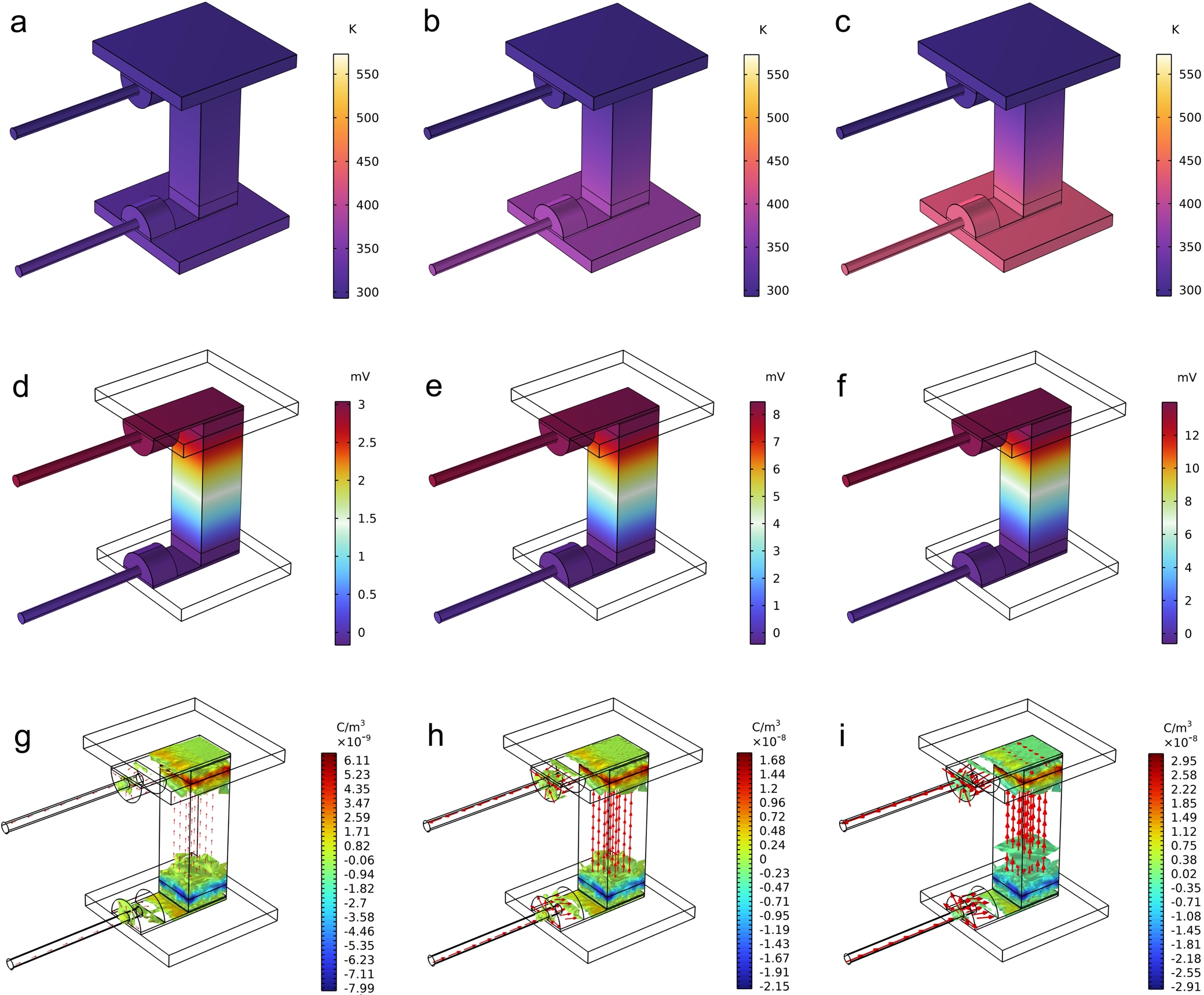


**Fig. S9 a–c,** Temperature distributions. **d–f,** Electric potential distributions. **g–i,** Current density distributions. The simulations correspond to temperature gradients (Δ*T*) of 30 K (**a**, **d**, **g**), 82 K (**b**, **e**, **h**), and 132 K (**c**, **f**, **i**), respectively.

**

**

**Fig. S10** a–d, Temperature-dependent characteristics of the thermoelectric module: internal resistance (*R*_in_) (**a**), output power (*P*_out_) (**b**), heat flow (*Q*_in_) (**c**), and conversion efficiency (*η*) (**d**). The plots display both experimental measurements and FEM simulation results. Thermal contact resistances were excluded from the simulation models due to the complexity of the multiple interfaces involved, specifically those between the high-temperature heat source and the Cu electrode, the Ni metallization layer and the thermoelectric materials, and the Cu electrode and the heat sink. Consequently, the discrepancy between the theoretical and measured results indicates the presence of residual thermal contact resistances that can be further minimized.

## Supplementary Tables

**Table S1 |** EDS results of the BST with 1.5 mol% PTO sample.

**
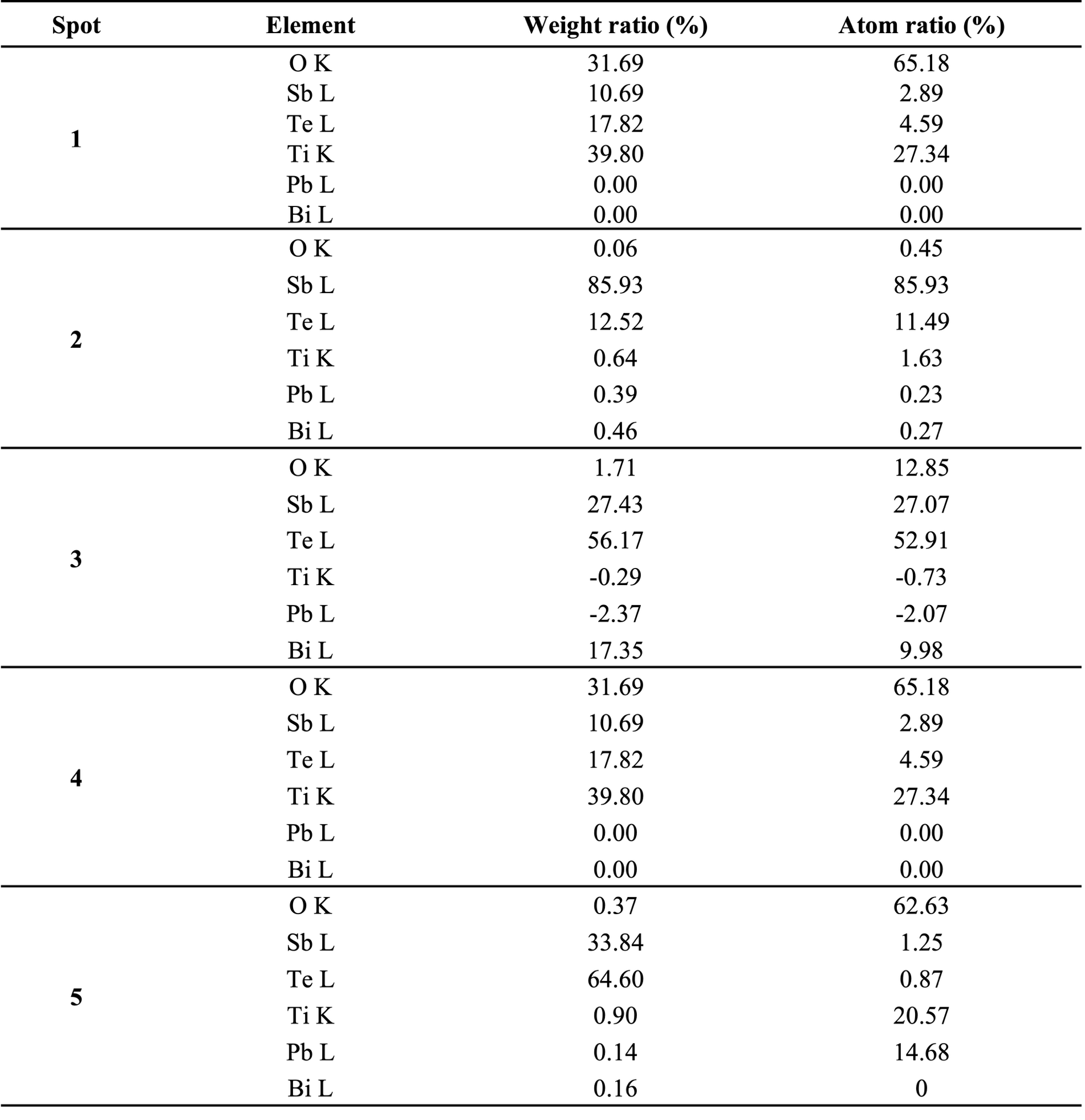
**

**Table S2 |** Parameters used for calculating thermoelectric properties based on the two-band Kane model.

| **Parameters** | **Values** |
| --- | --- |
| Combination of elastic constants *C*_l_ (Pa) | 4.57×10^10^ |
| Band gap *E*_g_ @300K (eV) | 0.156 |
| Band convergency of *N*_C_ and *N*_V_ | 6 |
| Ratio of the longitudinal to transverse band | 1 |
| effective mass *K*^*^ |  |
| Deformation potential of *E*_def_ (eV) | 47 (Fitted) |
| Density of states effective mass *m*^*^ | 0.045 (Cal.) |
| Average lattice thermal conductivity (W m^-1^ K^-1^) | 0.4985 (measured) |

**Table S3 |** Parameters used for calculating *κ_L_* and *κ_L,spectral_* considering various phonon scattering mechanisms.

| **Parameters** | **Values** |
| --- | --- |
| Lattice constant a (Å) | 10.061 |
| Average atomic mass *M* (Kg) | 2.223×10^-25^ |
| Average atomic mass volume *V*_0_ (m^3^) | 3.3943×10^-29^ |
| Longitudinal velocity *v*_L_(m/s) | 2884 (Exp.) |
| Transverse velocity *v*_T_ (m/s) | 1780 (Exp.) |
| Average sound velocity *v* (m/s) | 1963 (Exp.) |
| Debye temperature *θ*_D_ (K) | 181 (Cal.) |
| Poisson's ratio | 0.19^[12]^ |
| Grüneisen parameter *γ* | 1.49^[13]^ |
| Scattering parameter *Γ* (×10^-3^) | 4.378 (Fitted) |
| Deformation potential of *E*_def_ (eV) | 47 (Fitted) |
| Density of states effective mass *m*^*^ | 0.045 (Cal.) |
| Grain size *L* (μm) | 5.5 (Exp.) |
| Density of second phases | 9.833×10^22^ (Cal.) |

**Table S4 |** Initial values used for the full-parameter calculation of power generation.

| **Parameters** | **Values** |
| --- | --- |
| *T*_cold_ of power generation (K) | 298 |
| *CTE*_c_ (W m^-2^ K^-1^) | 1.2×10^4[14]^ |
| *CTE*_h_ (W m^-2^ K^-1^) | 6×10^3[14]^ |
| Leg height of PTO/BST-BTS module (mm) | 13.5 |
| *A*_p_ */ A*_n_ | 1.3 |
| *A*_p_ of p-type PTO/BST leg (mm^2^) | 3.2*3.7 |
| *A*_n_ of n-type BTS leg (mm^2^) | 3.3*2.7 |

## Supplementary References

[1] B. Qin, D. Wang, W. He, Y. Zhang, H. Wu, S. J. Pennycook, L.-D. Zhao, *J. Am. Chem. Soc.* **2018**, *141*, 1141.

[2] J. Callaway, H. C. von Baeyer, *Phys. Rev.* **1960**, *120*, 1149.

[3] W. He, D. Wang, H. Wu, Y. Xiao, Y. Zhang, D. He, Y. Feng, Y.-J. Hao, J.-F. Dong, R. Chetty, L. Hao, D. Chen, J. Qin, Q. Yang, X. Li, J.-M. Song, Y. Zhu, W. Xu, C. Niu, X. Li, G. Wang, C. Liu, M. Ohta, S. J. Pennycook, J. He, J.-F. Li, L.-D. Zhao, *Science* **2019**, *365*, 1418.

[4] C. L. Wan, W. Pan, Q. Xu, Y. X. Qin, J. D. Wang, Z. X. Qu, M. H. Fang, *Phys. Rev. B* **2006**, *74*, 144109.

[5] K. Kurosaki, A. Kosuga, H. Muta, M. Uno, S. Yamanaka, *Appl. Phys. Lett.* **2005**, *87*, 061919.

[6] D. Bessas, I. Sergueev, H.-C. Wille, J. Perßon, D. Ebling, R. P. Hermann, *Phys. Rev. B* **2012**, *86*, 224301.

[7] M. Hong, Z. Chen, L. Yang, Y. Zou, M. S. Dargusch, H. Wang, J. Zou, *Adv. Mater.* **2018**, *30*, 1705942.

[8] J. Callaway, *Phys. Rev.* **1959**, *113*, 1046.

[9] K. Kim, G. Kim, H. Lee, K. H. Lee, W. Lee, *Scr. Mater.* **2018**, *145*, 41.

[10] C.-C. Lin, D. Ginting, R. Lydia, M. H. Lee, J.-S. Rhyee, *J. Alloys Compd.* **2016**, *671*, 538.

[11] B. Zhu, Q. Zhai, H. Dai, W. Zhou, R. Xiong, Z. Liu, R. Huang, C. Li, W. Cao, Y. Zheng, Z. Wang, *Mater. Today Energy* **2026**, *56*, 102213.

[12] H. Zhang, D. T. Yimam, S. de Graaf, J. Momand, P. A. Vermeulen, Y. Wei, B. Noheda, B. J. Kooi, *ACS Nano* **2021**, *15*, 2869.

[13] J. O. Jenkins, J. A. Rayne, R. W. Ure, *Phys. Rev. B* **1972**, *5*, 3171.

[14] T. Xing, Q. Song, P. Qiu, Q. Zhang, M. Gu, X. Xia, J. Liao, X. Shi, L. Chen, *Energy Environ. Sci.* **2021**, *14*, 995.
